# Editorial: Mental health challenges in health professions education

**DOI:** 10.3389/fpsyt.2026.1798884

**Published:** 2026-02-06

**Authors:** Rong Wang, Xiaoming Xu

**Affiliations:** 1 Department of Physiology and Pathophysiology, School of Basic Medical Sciences, Shandong University, Jinan, China; 2 Center for Teaching and Learning in Medical Education, The Fourth Affiliated Hospital of School of Medicine, International School of Medicine, and International Institutes of Medicine, Zhejiang University, Yiwu, China

**Keywords:** educational interventions, health professions education, mental health challenges, student psychological distress, systemic factors

Mental health challenges among learners in the health professions have become a global and increasingly visible concern. Health professions education is widely recognized as intellectually demanding, emotionally taxing, and structurally intensive, exposing students to sustained academic pressure, high-stakes assessment, and early professional responsibility. Against this background, the Mental Health Challenges in Health Professions Education was conceived to bring together empirical evidence that not only documents the burden of psychological distress but also explores its antecedents, correlates, and potential educational responses within diverse sociocultural contexts.

The articles included in this Research Topic collectively underscore that mental health challenges in health professions education are neither isolated nor incidental. Instead, they emerge from the complex interaction between individual characteristics, learning environments, and broader systemic conditions. By drawing on studies conducted across multiple countries and educational settings, this topic highlights both the universality of mental health concerns among health professions students and the contextual factors that shape their manifestation.

## The burden of psychological distress during training

Several contributions provide compelling evidence of the substantial psychological burden experienced by students during health professions training. A cross-sectional study conducted among medical students in Greece reports high levels of burnout, emphasizing emotional exhaustion and depersonalization as prominent features during undergraduate medical education Angelopoulos, et al. These findings reinforce long-standing concerns that current training structures may inadvertently normalize chronic stress and fatigue rather than mitigate them.

Similarly, a large cross-sectional study from China examines the current status of psychological health among medical students and demonstrates a clear association between poorer mental health and lower academic performance Chunhong, et al. This linkage is particularly important for educators and institutional leaders, as it challenges the assumption that psychological distress is a private or peripheral issue. Instead, mental health emerges as a core educational variable with direct implications for learning outcomes, progression, and professional development.

Taken together, these studies suggest that mental health challenges are not merely by-products of rigorous training but may actively undermine educational goals if left unaddressed. They also highlight the need to move beyond prevalence estimates toward a deeper understanding of how educational structures and assessment cultures contribute to sustained distress.

## Extreme outcomes and hidden risk factors

While distress and burnout are often discussed as common experiences, one article in this Research Topic draws attention to more severe and potentially irreversible outcomes. A cross-sectional cohort study from Mexico explores hidden risk factors associated with suicidal behavior among medical students Martinez-Fierro, et al. The findings point to a constellation of psychological, social, and academic stressors that may accumulate over time, increasing vulnerability to self-harm.

The inclusion of this work is particularly important, as it reminds educators and policymakers that mental health challenges in health professions education exist along a continuum. For a subset of students, unresolved distress may escalate into crises with profound personal and institutional consequences. This study underscores the ethical responsibility of training institutions to recognize early warning signs, reduce stigma around help-seeking, and establish accessible, confidential support systems.

## Interpersonal competencies and adaptive capacity

Beyond documenting distress, articles within this Research Topic also considered individual attributes that may influence students’ capacity to navigate challenging learning environments. One study examines the association between empathy and assertiveness among undergraduate medical students in Punjab Fatima, et al. These interpersonal competencies are central to professional identity formation and clinical practice, but they may also function as protective or risk factors in relation to mental health.

The findings suggest that students’ interpersonal styles are meaningfully linked to how they engage with peers, patients, and faculty, and potentially to how they cope with stress and conflict. From an educational perspective, this highlights the importance of curricula that explicitly support the development of communication skills, self-advocacy, and emotional awareness, rather than assuming these competencies will emerge spontaneously through clinical exposure.

## Educational interventions and mental health literacy

Importantly, the articles within this Research Topic also points toward solutions. An innovative educational intervention, Psycheutopia, is presented as a structured program designed to enhance mental health literacy among medical students Jabari, et al. This work demonstrates how targeted educational initiatives can improve students’ understanding of mental health, reduce misconceptions, and promote more adaptive attitudes toward psychological well-being.

The inclusion of an intervention-focused study signals a shift from reactive to proactive approaches in health professions education. Rather than relying solely on counseling services that intervene after distress has become entrenched, mental health literacy programs aim to equip students with knowledge and skills that support early recognition, self-care, and peer support. Such approaches align well with contemporary calls for whole-of-curriculum strategies to promote well-being.

Collectively, these studies suggest that mental health challenges in health professions education should be understood as systemic rather than individual failings. Psychological distress, burnout, and extreme outcomes such as suicidal behavior are shaped by assessment practices, workload expectations, learning climates, and institutional cultures. At the same time, individual characteristics and competencies can either buffer or exacerbate these pressures.

Future research should continue to integrate descriptive epidemiology with intervention studies, while also attending to contextual and cultural differences across educational systems. For educators and institutional leaders, the evidence presented here reinforces the need for multi-level strategies that combine curriculum design, faculty development, student support services, and policy reform. Creating learning environments that are both academically rigorous and psychologically sustainable is not only a matter of student welfare but also a prerequisite for training competent, compassionate, and resilient health professionals.

In summary, this Research Topic advances the field by offering a nuanced and internationally grounded perspective on mental health challenges in health professions education. By synthesizing evidence on prevalence, risk factors, interpersonal competencies, and educational interventions, the included articles collectively contribute to a more holistic understanding of how mental health can be protected and promoted within the training of future health professionals.

